# Invasion of *Wolbachia* into *Anopheles* and Other Insect Germlines in an *Ex vivo* Organ Culture System

**DOI:** 10.1371/journal.pone.0036277

**Published:** 2012-04-30

**Authors:** Grant L. Hughes, Andrew D. Pike, Ping Xue, Jason L. Rasgon

**Affiliations:** 1 The Department of Entomology, Center for Infectious Disease Dynamics and Huck Institutes of the Life Sciences, Pennsylvania State University, University Park, Pennsylvania, United States of America; 2 The W. Harry Feinstone Department of Molecular Microbiology and Immunology, Bloomberg School of Public Health, Johns Hopkins University, Baltimore, Maryland, United States of America; Centro de Pesquisas René Rachou, Brazil

## Abstract

The common bacterial endosymbiont *Wolbachia* manipulates its host's reproduction to promote its own maternal transmission, and can interfere with pathogen development in many insects making it an attractive agent for the control of arthropod-borne disease. However, many important species, including *Anopheles* mosquitoes, are uninfected. *Wolbachia* can be artificially transferred between insects in the laboratory but this can be a laborious and sometimes fruitless process. We used a simple *ex vivo* culturing technique to assess the suitability of *Wolbachia*-host germline associations. *Wolbachia* infects the dissected germline tissue of multiple insect species when the host tissue and bacteria are cultured together. Ovary and testis infection occurs in a density-dependent manner. *Wolbachia* strains are more capable of invading the germline of their native or closely related rather than divergent hosts. The ability of *Wolbachia* to associate with the germline of novel hosts is crucial for the development of stably-transinfected insect lines. Rapid assessment of the suitability of a strain-host combination prior to transinfection may dictate use of a particular *Wolbachia* strain. Furthermore, the cultured germline tissues of two major Anopheline vectors of *Plasmodium* parasites are susceptible to *Wolbachia* infection. This finding further enhances the prospect of using *Wolbachia* for the biological control of malaria.

## Introduction

*Wolbachia* are intracellular α-proteobacteria that infect approximately two-thirds of insect species along with numerous other arthropods [1], [2]. Generally classified as reproductive parasites, *Wolbachia* are able to manipulate their hosts reproduction in various ways [3]. These reproductive manipulations exploit the maternal transmission route of the bacteria to the progeny of the host, facilitating the spread of *Wolbachia* into the host population. In addition to maternal transmission, the incongruence between the phylogenies of *Wolbachia* and their hosts suggests horizontal transmission has occurred repeatedly over evolutionary time, which has extended the host range of the bacteria [4], [5], [6]. The ability of *Wolbachia* to invade new species has been reproduced in the laboratory, with artificial transfers into novel insect species. Despite transinfection of *Wolbachia* into many new insect species, it is uncertain why some species are capable of infection yet others are seemingly not.

For either horizontal transmission or transinfection to occur, *Wolbachia* must establish in the female germline to allow the bacteria to be transmitted to the next generation. When artificially transferred into a new host, *Wolbachia* are usually microinjected into the preblastoderm embryo, a scenario that is unlikely to occur in nature. However, in experiments that more closely mimic potential natural routes of horizontal transfer, *Wolbachia* has been shifted between insects by adult injection, hemolymph transfer and co-rearing [7], [8], [9]. When injected into uninfected *Drosophila*, *Wolbachia* migrate across numerous tissues to establish in the stem cell niche of the ovary [7]. Hemolymph transfer between infected and uninfected pillbugs (*Armadillidium vulgare*, *Porcellionides pruinosus* and *Chaetophiliscia elongate*) resulted in stable intraspecific transfer of *Wolbachia* and manipulation of host reproductive [8].

The capacity for *Wolbachia* to spread though host populations and interfere with pathogen development in insects makes them attractive agents for the control of vector-borne diseases. Currently, *Wolbachia* is being investigated to control malaria, a devastating disease transmitted by *Anopheles* mosquitoes. *Wolbachia* is capable of infecting *Anopheles* cells *in vitro* and *Anopheles* somatic tissues *in vivo*, and influences the expression of numerous host genes [10], [11], [12]. After injection into adult *Anopheles* mosquitoes, *Wolbachia* influences *Plasmodium* levels, with *w*AlbB and *w*MelPop reducing *P. falciparum* intensity [10]. Interestingly, somatic infection of *An. gambiae* with the *w*AlbB strains enhances oocyst density of *P. berghei*, the model murine malaria species [13]. However, all *Anopheles* mosquitoes lack *Wolbachia* infection in the wild and despite numerous attempts seem impervious to stable transinfection in the lab [14], [15]. To increase the probability of infecting *Anopheles* mosquitoes, a variety of combinations of *Wolbachia* strains and *Anopheles* species should be assessed to determine which pairing is most likely to be successful.

Establishing stable *Wolbachia* infections in novel hosts by embryonic or adult microinjection is a very labor-intensive process with a high failure rate. The development of a rapid assay to assess which *Wolbachia* strains are most effective at invading the insect germline would greatly facilitate the generation of novel stable *Wolbachia* infections in insect hosts of interest. Various mosquito tissues have been cultured in an *ex vivo* setting using cell culture media [16], [17]. Fat body and ovarian culture has been used to dissect mosquito physiological responses [18], [19], [20]. As such, *ex vivo* mosquito organ culture provides a controlled tractable system to assess specific experimental treatments on insect tissues. Here, we utilized *ex vivo* tissue culture to develop a simple, rapid, assay to assess germline infectivity in novel *Wolbachia*-host combinations.

## Results

### *Wolbachia* infects the germline of multiple insect species in culture

**Figure 1:**
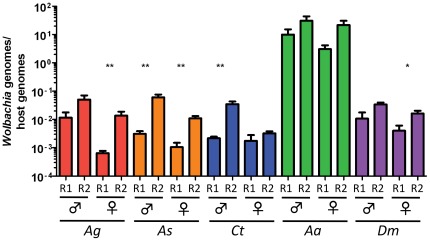
*Wolbachia* density (*Wolbachia* genomes/host genome) in the germline (ovaries and testes) of *An. gambiae*, *An. stephensi*, *C. tarsalis*, *Ae. aegypti* and *D. melanogaster* 2 days post infection. Two replicate experiments were completed to infect the germline of insects with wAlbB 8.5×10^4^ live cells inoculated into each well for replicate 1 (R1), while 5.5×10^5^ live cells were added to each well in replicate 2 (R2). For each replicate, five pools, each containing five pairs of ovaries or testes, were evaluated for *Wolbachia* density. Asterisk(s) denote significance (** P<0.01, * P<0.05). Error bars indicated SEM.

The host species chosen for *Wolbachia* invasion experiments varied in their natural *Wolbachia* infection status; naturally infected and cured (*D. melanogaster*), uninfected yet empirically susceptible to transinfection (*Ae. aegypti*), or uninfected (*An. gambiae, An. stephensi, C. tarsalis*). qPCR analysis comparing the number of *Wolbachia* genomes normalized to host genome copy number found that both the male and female germline tissues of all five insect species were susceptible to *Wolbachia* infection. There was a positive correlation between inoculum titer and *Wolbachia* germline infection density, indicating that *ex vivo* infection occurred in a density-dependent manner. In five of the ten infection experiments, significantly higher *Wolbachia* densities were observed in ovaries inoculated with a higher titer of bacteria. In each of the five other cases, there was a trend towards higher bacterial densities when a greater amount of *Wolbachia* was used to inoculate the germline tissue, though the differences were not statistically significant (Figure 1).

**Figure 2:**
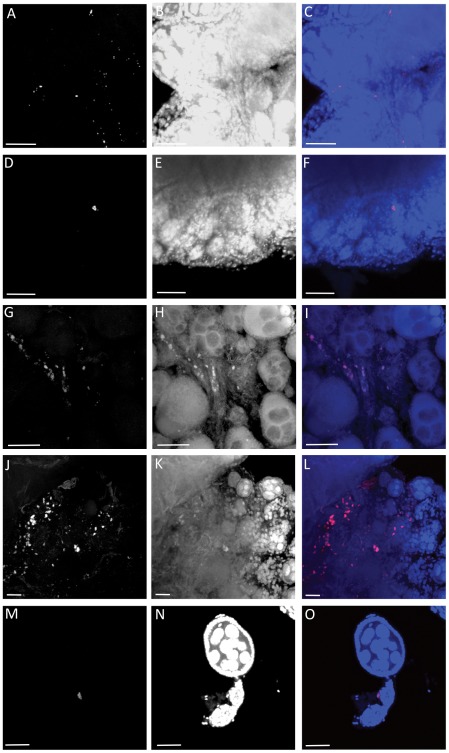
FISH images of *w*AlbB infection in insect ovaries captured using a confocal microscope for *An. gambiae* (A–C), *An. stephensi* (D–F), *Ae. aegypti* (G–H) *C. tarsalis* (J–L) and *D. melanogaster* (M–O). Images in the left column are the red channel only (*Wolbachia*), images in the center column are blue channel only (host nuclei) and images in the right column are the merged image (Red: *Wolbachia*, Blue: host nuclei) The Z-stacks of the ovaries are available as supplementary videos (Movie S1, S2, S3, S4, S5). The scale bar represents 20 µm. Images are compiled from compression of Z stack with a depth of 5 µm.

To examine the localization of the infection and confirm that qPCR results were not due to surface contamination by *Wolbachia*, we performed fluorescence *in situ* hybridization (FISH) on *w*AlbB-infected ovaries followed by confocal microscopy. *Wolbachia* was visually detected in the ovaries of all five species (Figure 2, Movies S1, S2, S3, S4, and S5). Most of the infection was observed in the ovarian follicular epithelium, however fluorescent signal was also seen within the *An. gambiae* ovarian follicle (Figure S1). Corroborating the qPCR results, a much more intense *w*AlbB infection was seen in *Ae. aegypti* ovaries compared to the other four insect species (Figure 2).

### *Wolbachia* reaches higher infection titer in native hosts and close relatives

**Figure 3:**
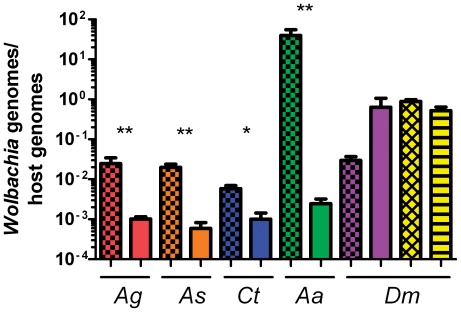
Comparison of the density (*Wolbachia* genomes/host genome) of multiple *Wolbachia* strains cultured in the *ex vivo* setting. *Wolbachia* strains: *w*AlbB (checkered) and *w*MelPop (solid) in ovaries of *An. gambiae*, *An. stephensi*, *C. tarsalis Ae. aegypti*, and *D. melanogaster* 2 days post infection. Ovaries naturally infected with *w*MelPop from *D. melanogaster* were cultured *ex vivo* for 2 days (yellow hatched) and are compared to the density in the fly (yellow horizontal lines). As the density of *Wolbachia* used as the inoculum for uninfected ovaries differed between strains, the final *Wolbachia* genomes:host genomes value were normalized to 10^6^ bacterial cells to compare between strains. The *w*MelPop strain infects *D. melanogaster* ovaries approximately 500 to 3000 fold higher, while *w*AlbB infects *Ae. aegypti* ovaries 1000 to 7000 fold greater than the other insect species. Asterisk(s) denote significance (** P<0.01, * P<0.05). There was no significant difference in *w*MelPop titer after two days of *ex vivo* culture compared to *in vivo*. For each insect species and *Wolbachia* strain, five pools, each containing five pairs of ovaries, were evaluated for *Wolbachia* density. Error bars indicated SEM.

Analysis by qPCR shows that both the ovaries and testes of *Ae. aegypti* are far more susceptible to infection with *w*AlbB (from the closely related *Ae. albopictus*) than the other species tested (Figure 1), reaching titers 1000–7000 fold higher than other species (Figure 3) (Kruskal-Wallis test P<0.02, Dunn's test shown in Table S1). Similarly, the *w*MelPop strain from *D. melanogaster* infected fly ovaries at a density approximately 500 to 3000 fold higher than the ovaries of other mosquito species (Figure 3) (Kruskal-Wallis P<0.02, Dunn's test shown in Table S1). The *w*MelPop strain, which has been transinfected into *Ae. aegypti* mosquitoes [21], infects the *Ae. aegypti* germline in the *ex vivo* assay at a higher density than both *Anopheles* mosquito species (2.4 times greater than *An. gambiae* (P<0.03); 4.2 times greater than *An. stephensi* (P<0.03)) (Figure 3). We also cultured ovaries from a naturally *w*MelPop-infected host using the *ex vivo* system. Infected ovaries from 20-day-old *D. melanogaster* were cultured and ovarian *Wolbachia* titer was compared to levels in ovaries immediately dissected from the fly. There was no significant difference in the *w*MelPop titer *in vivo* compared to *ex vivo*, nor between *Wolbachia* titer of naturally infected ovaries after *ex vivo* culture and titer in fly ovaries which were infected using the *ex vivo* system (Figure 3).

### *w*AlbB infects *Anopheles* germline more efficiently than *w*MelPop

Our data suggest that *Anopheles* ovaries are more susceptible to infection with the *w*AlbB strain than the *w*MelPop strain. After normalizing the densities of *Wolbachia* in the inoculum, the *w*AlbB strain infected the *Anopheles* germline at a greater density (24 times for *An. gambiae* (P<0.008) and 34 times for *An. stephensi* (P<0.008) than the *w*MelPop strain (Figure 3).

### *Wolbachia* infects, but does not replicate in, cultured germline tissue

**Figure 4:**
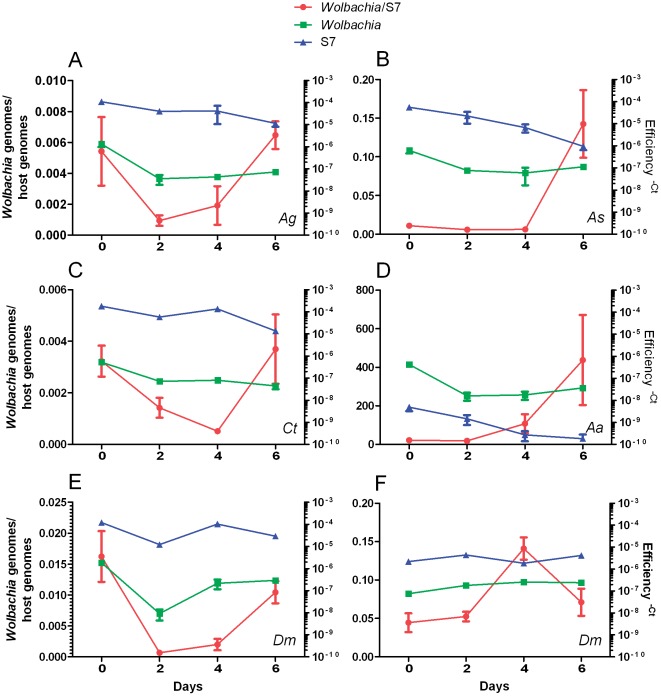
Density of *Wolbachia* in insect ovaries measured over time. The left axis indicates the ratio of *Wolbachia* genomes/host genome (red line). The right axis indicates the fold change for *Wolbachia* (green) and host (blue) single copy gene for each species: *An. gambiae* (A; *Ag*), *An. stephensi* (B; *As*), *C. tarsalis* (C; *Ct*) *Ae. aegypti* (D; *Aa*) and *D. melanogaster* (E; *Dm*) infected with *w*AlbB. *D. melanogaster* ovaries naturally infected with *w*MelPop were also cultured (F; *Dm*). For each insect species infected with *w*AlbB, three pools, each containing five pairs of ovaries, were evaluated for *Wolbachia* density. Four pools each containing five pairs of ovaries were completed for naturally infected *D. melanogaster* ovaries. Error bars indicate SEM.

**Figure 5:**
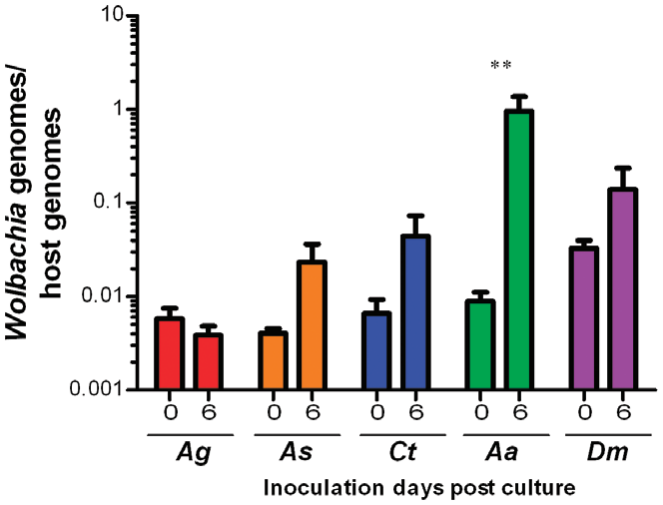
The effect of culturing before inoculation of *Wolbachia* on ovary density. Ovaries were inoculated with *Wolbachia* immediately after culture (day 0) or 6 days post culture for *An. gambiae* (Ag), *An. stephensi* (As), *C. tarsalis* (Ct), *Ae. aegypti* (Aa) and *D. melanogaster* (Dm). Two days post-infection, the density of *Wolbachia* was compared in these two treatments. Density of *Wolbachia* in ovaries was significantly different (P<0.007) between the *Ae. aegypti* day 0 and day 6 treatments. For each insect species, five pools, each containing five pairs of ovaries, were evaluated for *Wolbachia* density. Error bars indicate SEM.

To determine if *Wolbachia* can replicate in cultured ovaries, we assayed bacterial density in the cultured host tissues at various times post-infection. For some species, we saw an initial drop in titer after the removal of *Wolbachia* from the media, but in all species there was an apparent increase in the ratio of *Wolbachia* to host genome copies by six days post inoculation (Figure 4). However, these differences were not statistically significant. Further examination of the results showed that the elevated density at latter time points was due mainly to a reduction in the number of host genomes rather than an increase in *Wolbachia* genomes. To confirm this, we completed another two experiments. First, we cultured naturally infected ovaries from 1–3 day old *D. melanogaster* and measured the changes in *Wolbachia* density over time. The variation in the S7 gene had a major bearing on the ratio of *Wolbachia* genomes to host genomes (Figure 4F). Second, we cultured host tissue for 6 days prior to addition of *Wolbachia* and compared the observed densities to those of ovaries inoculated with the same titer of *Wolbachia* immediately after dissection. Delaying the inoculation resulted in significantly higher *Wolbachia*:host gene ratios for, *Ae. aegypti* (P<0.007) (Figure 5), while in three other species there was a general trend towards higher values, indicating that for most species, and particularly *Aedes*, host degradation was responsible for the increase in relative *Wolbachia* density observed in the time course experiments. Taken together, these data suggest *Wolbachia* is not significantly replicating in ovaries in an *ex vivo* environment.

## Discussion

In order to be maternally transmitted after horizontal transfer, *Wolbachia* must first invade the host ovarian tissues. Previously, we reported that *Wolbachia* is capable of replicating within *Anopheles* cells *in vitro* and profoundly influences host gene expression [11], [12]. After microinjection of the *w*MelPop strain *Wolbachia* into adult female *Anopheles* mosquitoes, the bacteria was noticeably absent from the ovarian tissue, despite dissemination to and replication in numerous other host tissues [10].

Here, we show that *Anopheles* and other insect germlines are amenable to *Wolbachia* infection in an *ex vivo* organ culture system. After two days of co-culture, ovaries became infected with *Wolbachia*, predominantly in the ovarian follicular epithelium, though there was evidence of *Wolbachia* residing in the *An. gambiae* oocyte as well. It is possible that, in order to migrate through follicular epithelium cells, *Wolbachia* may require the cells to be metabolically active, which may not occur under *ex vivo* culture conditions. After injection of *w*Mel into uninfected *Drosophila*, the bacteria were seen to infect the follicular cells at approximately 6–8 days and the germline tissue after approximately 15 days [7]. Our observation of shorter invasion times most likely results from the ability of *Wolbachia* to directly access the germline tissue without the need to migrate through somatic tissue or contend with the host immune response.

We then compared the infection properties of two divergent strains of *Wolbachia* (*w*AlbB from *Ae. albopictus* and *w*MelPop from *D. melanogaster*). The ability of *Wolbachia* to invade it's native host (*w*MelPop and *D. melanogaster*) and closely related hosts (*w*AlbB and *Ae. aegypti*) at a higher density suggests that these relationships have developed in close association and are more stable than more disparate associations. Additionally, levels of *w*MelPop from naturally infected ovaries cultured in the *ex vivo* system for 2 days were similar to both the density of *w*MelPop in naturally-infected fly ovaries and the density of *w*MelPop that had invaded uninfected fly ovaries in the *ex vivo* system, suggesting that the density of infection in the *ex vivo* system has biological relevance. Taken together, these results validate the *ex vivo* culturing technique as a tool to assess *Wolbachia*-host associations.

To ascertain if *ex vivo* culture can be used to adapt *Wolbachia* to the *Anopheles* germline environment, we assayed *Wolbachia* density over time to determine if the bacteria replicates in cultured ovarian tissue. Although the ratio of *Wolbachia* to host genomes increased in all species over time, further analysis of the number of copies of *Wolbachia* and host reference genes implicated degradation of the host cells as the main cause of the observed increase in *Wolbachia* density. In some species, there is an initial decline in *Wolbachia* 2 dpi. This may indicate that bacteria that were bound to the ovary at 0 dpi failed to enter and consequently, later time points had lower *Wolbachia* titers. Culturing naturally infected ovaries gave similar results to infection *ex vivo* in that the host gene markedly influenced the density (ratio of *Wolbachia* genomes to host genomes). Delaying addition of the *Wolbachia* inoculum to the cultured *Ae. aegypti* ovaries significantly increased the titer, suggesting for this species that host degradation is responsible for the increase in *Wolbachia* density over time, rather than replication of the bacteria in the germline tissue. While it has been reported that ovaries can be cultured using similar techniques for over 2 months [17], it is likely that the tissue is not metabolically active. Host derived factors may therefore be absent which could be critical for *Wolbachia* replication. In naturally infected *Drosophila* oocytes, *Wolbachia* replication has been observed during the period of ovarian development approximately mid-oogenesis [22]. The lack of an active cell cycle may be a reason for the observed lack of replication in cultured ovaries. As the bacteria do not seem to be replicating in the host tissue, we presume that this approach is not suitable to pre-adapt *Wolbachia* to the *Anopheles* germline environment.

If these *ex vivo* results are representative of the live mosquito, they suggest that *Wolbachia* is able to successfully infect the ovaries of *Anopheles*. However, stable introduction of *Wolbachia* into these important vectors has not been accomplished, nor has infection been observed in the wild. Previous studies have indicated that *Wolbachia* are able to both invade and reproduce in *Anopheles* cells in culture and adult somatic tissues, but have not reported invasion into reproductive tissues [10], [11]. Here, we show that the reproductive tissues can be infected when other host factors are absent, but that *Wolbachia* do not reproduce under these conditions. Therefore, the question as to why *Anopheles* species remain uninfected with *Wolbachia* remains. Possible explanations include a lack of reproduction in *Anopheles* ovaries under natural conditions, negative interactions between the bacteria and the host, factors blocking invasion of the ovaries in adult mosquitoes and many others, requiring further elucidation if *Wolbachia* is to be used for malaria control.

The infection of *ex vivo* cultured insect ovaries with *Wolbachia* can be used as a tool for the assessment of the suitability of various *Wolbachia* strains to infect a given species of interest. By testing the ability of different *Wolbachia* strains to invade the host germline in culture, the strain with the greatest ability to do so can be chosen for transinfection. Given the similar horizontal infection routes, data gleaned from this approach may be more useful for assessment of adult transinfections. However, in general, affinity for the germline is critical for any novel *Wolbachia*-host association regardless of whether the transinfection is by adult or embryonic microinjection. This system can also be used to investigate the interactions between multiple *Wolbachia* strains infecting the same host and to examine the interactions between naturally infected hosts and their *Wolbachia*. Finally, *ex vivo* culture provides a tractable method by which the effects of various treatments on the ability of *Wolbachia* to invade host germline tissues can be investigated.

For *Wolbachia* to be used for vector control in novel hosts, stably transinfected lines must be generated, which requires that the bacteria successfully invade the host germline tissue. Here, we demonstrate a new method for quickly evaluating the suitability of a given *Wolbachia* strain to invade the reproductive tissues of an insect host. Using this method, we show that *Wolbachia* is able to infect the ovaries of both *An. gambiae* and *An. stephensi*, two important vectors of human malaria parasites. Our results also indicate that *Wolbachia* invasion occurs in a density dependent manner and that *Wolbachia* are able to more efficiently infect the tissues of their natural host or closely related species than those of more distantly related insects, providing evidence for the adaptation of *Wolbachia* to their hosts during long term association. This study provides support for the possibility that *Wolbachia* can infect *Anopheles* mosquitoes, and therefore be useful in the control of malaria.

## Materials and Methods

### *Ex vivo* culture of insect germline tissues

Germline tissues (ovaries and testes) were dissected from two to three days old *Anopheles gambiae* (Keele strain), *An. stephensi* (Liston strain), *Culex tarsalis* (KNWR strain), *Aedes aegypti* (Rockefeller strain) and *Drosophila melanogaster* (*w* ^1118^ flies previously treated with tetracycline to remove the natural *Wolbachia* infection) in PBS using a dissecting microscope (Olympus). For culture experiments that used naturally infected ovaries, untreated *D. melanogaster* (*w* ^1118^ infected with *w*MelPop) were used. Prior to dissection, insects were surface sterilized with 100% ethanol for five minutes and rinsed in sterile PBS. After dissection, ovaries or testes were briefly washed with 100% ethanol and rinsed in Schneider's media with 10% fetal bovine serum (FBS) (Invitrogen), 50 µg/ml kanamycin (Sigma), 10 units/mL penicillin and 10 µg/ml streptomycin (Invitrogen). *Wolbachia* replication dynamics are not affected by kanamycin, penicillin or streptomycin [23], [24]. Five ovaries or testes were placed within a single cell culture insert (Millipore). Five replicates were completed for infection experiments, while three replicates were done for time course (replication) assays. Under sterile conditions, germline tissues were washed three times with 1 mL of fresh Schneider's media then incubated at room temperature in 1 mL of Schneider's media with 10% FBS, 50 µg/ml kanamycin (Sigma), 10 units/mL penicillin and 10 µg/ml streptomycin (Invitrogen).

### *Wolbachia* infection

The *w*AlbB and *w*MelPop strains of *Wolbachia* were purified from infected Sua5B [25] (provided by M. Jacobs-Lorena) and Mos55 [26] (provided by S. O'Neill) cells, respectively, according to previously published procedures [27]. Purified *Wolbachia* were stained using the Live/Dead BacLight Bacterial Viability Kit (Invitrogen) and counted using a hemocytometer under a fluorescence microscope (Olympus) to determine the density of viable cells. One microliter of purified *Wolbachia*, with a known density, was then added to each tissue culture well containing ovaries or testes. When naturally infected ovaries were assessed, purified *Wolbachia* was not added to the culture.

### Quantitative real-time PCR (qPCR)

After two days in culture, germline tissues were removed from the culture media, washed thoroughly 3 times with fresh Schneider's media before DNA was extracted using the QIAamp DNA Micro Kit (Qiagen). This DNA was used as template for qPCR to estimate the density of *Wolbachia* in cultured ovaries or testes. *w*AlbB was detected using primers GF and BR that amplify the *wsp* gene [10] while *w*MelPop was detected with primers amplifying the single copy WD_0550 gene [28]. *Wolbachia* densities were normalized to single-copy host genes. For *An. gambiae*, *An. stephensi* and *Ae. aegypti*, primers amplifying the S7 rRNA gene were used [29], [30], while primers which amplify a segment of the actin gene were used for *C. tarsalis* [31]. *D. melanogaster* S7 primers were designed using Primer3 [32]. Primer sequences are found in table S2. All qPCR was performed on a Rotor Gene Q (Qiagen) using the Rotor Gene SYBR Green PCR Kit (Qiagen) using manufacturers recommended settings. Each reaction contained DNA extracted from five ovaries or testes and was performed in duplicate. A third technical replicated was completed when there was a discrepancy between the replicates. *Wolbachia* to host genome ratios were calculated using Qgene [33]. Changes in individual *Wolbachia* or host gene values were estimated by raising the reaction efficiency to the exponent power of the negative threshold cycle (E^−Ct^) as previously described [27]. For single comparisons between infected ovaries, data were analyzed using a Mann-Whitney U test, while multiple comparisons were performed using a Kruskal-Wallace test followed by a Dunn's Post-Hoc test.

### Fluorescence *in situ* hybridization

In order to ensure that qPCR results were not due to *Wolbachia* contaminating the surface of cultured organs, we used fluorescence *in situ* hybridization (FISH) to assess *Wolbachia* infection in mosquito tissues. After two days in culture, FISH was completed on ovary tissue according to previously published procedures [10] with slight modifications. Briefly, Schneider's media was removed from culture wells and ovaries were washed thoroughly three times with fresh Schneider's media, fixed in 4% paraformaldehyde in PBS for 20 minutes then moved to 6% hydrogen peroxide in ethanol for 4 days to minimize autofluorescence. Ovaries were allowed to hybridize overnight in 1 ml of FISH hybridization buffer (50% formamide, 5× SSC, 200 g/liter dextran sulfate, 250 mg/liter poly(A), 250 mg/liter salmon sperm DNA, 250 mg/liter tRNA, 0.1 M dithiothreitol (DTT), 0.5× Denhardt's solution) with *Wolbachia* specific probes W1 and W2 labeled with a 5-prime rhodamine fluorophore [34]. After hybridization, ovaries underwent three successive washes in 1× SSC, 10 mM DTT and three times in 0.5× SSC, 10 mM DTT. Tissues were mounted on a slide with SlowFade Gold antifade reagent (Invitrogen) and counterstained with DAPI (Roche). Images were captured on an LSM 510 META confocal microscope (Zeiss) and compared to no probe controls. Images were processed using LSM image browsers (Zeiss) and Photoshop 7.0 (Adobe).

## Supporting Information

Movie S1 **Fluorescence** ***in situ*** **hybridization of** ***Wolbachia*** **infection in** ***An. gambiae*** **ovaries.** Serial confocal images of the Z-axis (Z-stack) of 23 sections (0.48 µm step size). Red is *Wolbachia*, blue is host nuclei (DAPI).
(MOV)

Movie S2 **Fluorescence** ***in situ*** **hybridization of** ***Wolbachia*** **infection in** ***An. stephensi*** **ovaries.** Serial confocal images of the Z-axis (Z-stack) of 15 sections (0.45 µm step size). Red is *Wolbachia*, blue is host nuclei (DAPI).
(MOV)

Movie S3 **Fluorescence** ***in situ*** **hybridization of** ***Wolbachia*** **infection in** ***C. tarsalis*** **ovaries.** Serial confocal images of the Z-axis (Z-stack) of 57 sections (0.49 µm step size). Red is *Wolbachia*, blue is host nuclei (DAPI).
(MOV)

Movie S4 **Fluorescence** ***in situ*** **hybridization of** ***Wolbachia*** **infection in** ***Ae. aegypti*** **ovaries.** Serial confocal images of the Z-axis (Z-stack) of 43 sections (0.49 µm step size). Red is *Wolbachia*, blue is host nuclei (DAPI).
(MOV)

Movie S5 **Fluorescence** ***in situ*** **hybridization of** ***Wolbachia*** **infection in** ***D. melanogaster*** **ovaries.** Serial confocal images of the Z-axis (Z-stack) of 25 sections (0.48 µm step size). Red is *Wolbachia*, blue is host nuclei (DAPI).
(MOV)

Figure S1 **Magnified image from a Z stack of** ***An. gambiae*** **(Movie S1) showing** ***Wolbachia*** **infection with the ovarian follicle.** Red is *Wolbachia*, blue is host nuclei (DAPI). The scale bar represents 10 µm.
(TIF)

Table S1 **Dunn's test for pairwise significance after a Kruskal-Wallis test comparing** ***Wolbachia*** **densities between all species (** **Figure 3** **).** Asterisks indicate significance after correcting for multiple comparisons using a false discovery rate of less than 5%.
(DOCX)

Table S2 **List of primers used for qPCR to estimate** ***Wolbachia*** **density.**
(DOCX)
