# Supplementary figures and images for: Invasion of Wolbachia into Anopheles and Other Insect Germlines in an Ex vivo Organ Culture System
Source: PLoS One. 2012 Apr 30;7(4):e36277. doi: 10.1371/journal.pone.0036277 (PMC3340357; doi:10.1371/journal.pone.0036277)

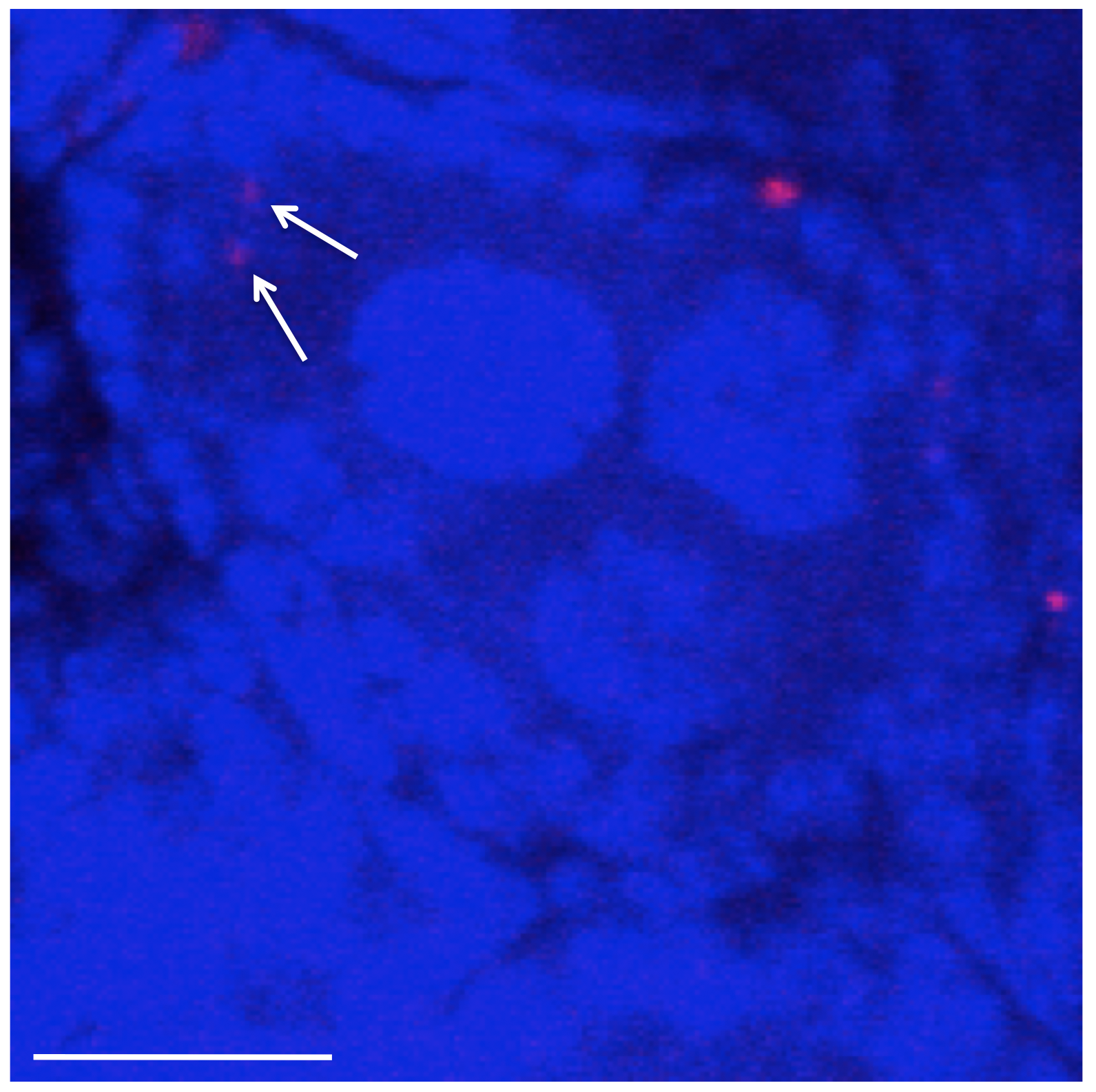

Supplement: Figure S1 — Magnified image from a Z stack of An. gambiae (Movie S1) showing Wolbachia infection with the ovarian follicle. Red is Wolbachia, blue is host nuclei (DAPI). The scale bar represents 10 µm. (TIF) [file pone.0036277.s006.tif]
