# Supplementary material for: Invasion of Wolbachia into Anopheles and Other Insect Germlines in an Ex vivo Organ Culture System
Source: PLoS One. 2012 Apr 30;7(4):e36277. doi: 10.1371/journal.pone.0036277 (PMC3340357; doi:10.1371/journal.pone.0036277)
Supplement: Table S1 — Dunn's test for pairwise significance after a Kruskal-Wallis test comparing Wolbachia densities between all species ( Figure 3 ). Asterisks indicate significance after correcting for multiple comparisons using a false discovery rate of less than 5%. (DOCX) [file pone.0036277.s007.docx]

| Species | P-value wAlbB | P-value wMelPop |
| --- | --- | --- |
| *An. gambiae* vs *An. stephensi* | 0.6873 | 0.3252 |
| *An. gambiae* vs *Ae. aegypti* | 0.0019 * | 0.1797 |
| *An. gambiae* vs *C. tarsalis* | 0.6228 | 0.6810 |
| *An. gambiae* vs *D. melanogaster* | 0.1284 | 0.0139 * |
| *An. stephensi* vs *Ae. aegypti* | 0.0065 * | 0.0200 * |
| *An. stephensi* vs *C. tarsalis* | 0.9287 | 0.6055 |
| *An. stephensi* vs *D. melanogaster* | 0.2636 | .00057 * |
| *Ae. aegypti* vs *C. tarsalis* | 0.0084 * | 0.937 |
| *Ae. aegypti* vs. *D. melanogaster* | 0.0958 | 0.2636 |
| *C. tarsalis* vs *D. melanogaster* | 0.3037 | 0.0063 * |
