# Supplementary material for: Invasion of Wolbachia into Anopheles and Other Insect Germlines in an Ex vivo Organ Culture System
Source: PLoS One. 2012 Apr 30;7(4):e36277. doi: 10.1371/journal.pone.0036277 (PMC3340357; doi:10.1371/journal.pone.0036277)
Supplement: Table S2 — List of primers used for qPCR to estimate Wolbachia density. (DOCX) [file pone.0036277.s008.docx]

| Primer | Sequence 5'-3' |
| --- | --- |
| AlbB-GF | GGTTTTGCTTATCAAGCAAAAG |
| AlbB-BR | GCGCTGTAAAGAACGTTGATC |
| MelPop WD_0550-F | CAGGAGTTGCTGTGGGTATATTAGC |
| MelPop WD_0550-R | TGCAGGTAATGCAGTAGCGTAAA |
| *Ae. aegypti* S7-F | GGGACAAATCGGCCAGGCTATC |
| *Ae. aegypti* S7-R | TCGTGGACGCTTCTGCTTGTTG |
| *Anopheles* S7-F | TCCTGGAGCTGGAGATGAAC |
| *Anopheles* S7-R | GACGGGTCTGTACCTTCTGG |
| *C. tarsalis* actin-F | GACTACCTGATGAAGATCCTGAC |
| *C. tarsalis* actin-R | GCACAGCTTTTCCTTGATGTCGC |
| *D. melanogaster* S7-F | CCATCCTTGAGGATCTGGTCTTC |
| *D. melanogaster* S7-R | GTCGACTTTGTGTTCAATGGTGG |
